# High-Performance Anti-Retransmission Deception Jamming Utilizing Range Direction Multiple Input and Multiple Output (MIMO) Synthetic Aperture Radar (SAR)

**DOI:** 10.3390/s17010123

**Published:** 2017-01-09

**Authors:** Ruijia Wang, Jie Chen, Xing Wang, Bing Sun

**Affiliations:** 1 Aeronautics and Astronautics Engineering College, Air Force Engineering University, Xi’an 710038, China; wangxing1099@sohu.com; 2 School of Electronics and Information Engineering, Beihang University, Beijing 100084, China; bingsun@buaa.edu.cn; 3 Collaborative Innovation Center of Geospatial Technology, Wuhan 430079, China

**Keywords:** SAR, MIMO, jamming suppression, range direction, sub-band synthesize

## Abstract

Retransmission deception jamming seriously degrades the Synthetic Aperture Radar (SAR) detection efficiency and can mislead SAR image interpretation by forming false targets. In order to suppress retransmission deception jamming, this paper proposes a novel multiple input and multiple output (MIMO) SAR structure range direction MIMO SAR, whose multiple channel antennas are vertical to the azimuth. First, based on the multiple channels of range direction MIMO SAR, the orthogonal frequency division multiplexing (OFDM) linear frequency modulation (LFM) signal was adopted as the transmission signal of each channel, which is defined as a sub-band signal. This sub-band signal corresponds to the transmission channel. Then, all of the sub-band signals are modulated with random initial phases and concurrently transmitted. The signal form is more complex and difficult to intercept. Next, the echoes of the sub-band signal are utilized to synthesize a wide band signal after preprocessing. The proposed method will increase the signal to interference ratio and peak amplitude ratio of the signal to resist retransmission deception jamming. Finally, well-focused SAR imagery is obtained using a conventional imaging method where the retransmission deception jamming strength is degraded and defocused. Simulations demonstrated the effectiveness of the proposed method.

## 1. Introduction

Synthetic aperture radar (SAR) has been widely used in civil exploration and military surveillance due to its all-weather, long-time and large-range detection capabilities [1]. However, rapidly developed electronic countermeasures (ECMs) can degrade the SAR imaging quality and system efficiency significantly [2,3,4]. The ECMs for SAR can be classified into barrage jamming and deception jamming, according to the signal characteristics and purpose of the ECM. Barrage jamming uses radio frequency noise with enough power to cover and corrupt the target signal. Deception jamming modulates fake target information on an intercepted signal retransmitted to the SAR via the direct-path or multipath routes [5]. Due to the high power consumption and easy identification and suppression, barrage jamming is less utilized compared to deception jamming. The false target formation by deception jamming will mislead the SAR image interpretation, which is difficult to recognize and mitigate, making deception jamming more flexible and efficient than barrage jamming.

As a result, anti-jamming and jamming suppression methods have become an appealing research topic in recent years [6,7,8]. Until now, most of the research and literature has been focused on the problem of narrow band interference (NBI) suppression, which is a classical unintentional interference caused by communication sources and broadcast and TV signals [9,10,11,12,13]. The relevant research on deception jamming suppression, wide band interference, is insufficient and not comprehensive. Miller proposed a parametric method based on building a mathematical model of the interference. Nevertheless, the effectiveness of jamming suppression is determined by the model’s degree of matching with the jamming. In [14], an adaptive two-dimensional filter, relying on estimating the statistical characteristics of the SAR image, was designed to suppress the NBI. Meanwhile, as for a classical non-parametric method, it is arduous to design an appropriate filter for the echo signal, which is not only blurred by NBI but also interfered with by wide band jamming. Methods such as Independent Component Analysis (ICA) and Independent Subspace Analysis (ISA) [12,13], were adopted for NBI suppression. These can extract the statistically independent jamming signal relying on the significant variance between jamming and target echoes in the data domain. ISA improves signal projection from the one-dimensional time domain to the two-dimensional time-frequency domain and reduces the constraints of ICA. However, when the jamming signal is not independent of the target echoes, the jamming suppression effect will be degraded. The aforementioned NBI suppression methods are not suitable for wideband interference, such as deception jamming. Tao et al. utilized the short-time Fourier transform (STFT) to convert the wideband interference (WBI) into a series of instantaneous NBI spectrum mitigation problems [15]. The premise of Tao’s method is that WBI has distinct energy variations in the time-localized spectra that can be captured by the STFT. However, deception jamming can intercept the SAR signal and retransmit it to the SAR without energy variations in localized time.

In essence, the main approach to deception jamming suppression is to use a complex signal during transmission and increase the signal to interference ratio (SIR) at the receiving terminal. The MIMO SAR [16,17] realizes this idea based on its multiple channel receiving and transmitting ability. In [18], Gebert proposed the structure of an azimuth MIMO SAR in order to improve the resolution. In [17], the OFDM signal, a typical independent waveform for MIMO radar, is used in MIMO SAR to reduce the range ambiguity. MIMO SAR can not only achieve high-resolution and wide-swath imaging but also has great potential in anti-jamming and jamming suppression. In [19], Rosenberg made use of the multiple channel signal receiving ability, a vital advantage of MIMO SAR, to cancel hot-clutter jamming, one type of multipath jamming. However, direct-path wide band jamming, such as retransmission deception jamming, was not mentioned.

To overcome the limitations of the aforementioned methods, we propose herein a novel MIMO structure, called range direction MIMO SAR, to suppress retransmission deception jamming. The multi-channel antenna of the MIMO SAR is arranged along the range direction. First, at the transmitting terminal, the orthogonal frequency division multiplexing (OFDM) LFM signal is adopted as the sub-band signal [20], which has a one-to-one correspondence with the transmitting channel of the antenna array. Then, random phases were modulated in each sub-band signal. After that, the modulated sub-band signal will be concurrently transmitted by the corresponding antenna. The concurrently transmitted sub-band signals will arrive at the jammer at the same time, which makes them hard to intercept and be recognized by the jammer. The jammer can only retransmit the signals with frequencies located in the jammer’s working frequency band. Hence, the jammer’s retransmission time delay will increase more than a Pulse Repetition Time (PRT), which means the phase of the retransmitted deceptive jamming signal will not match the target echo in the azimuth. Second, at the receiving terminal, the sub-band signals will be pre-processed, including the phase compensation, time shift and frequency shift. Then, the sub-band signals can be utilized to synthesize a wide band signal in the range direction. Meanwhile, part of the frequency band of the synthesized wide band signal will be blurred by the retransmission deceiving jamming. Finally, the traditional methods could be applied to get the SAR image of the synthesized wide band signal. After using the proposed methods, the jammer signal could not get the signal processing gain and will defocus in the SAR image because of the mismatch with the target echo. Meanwhile, the sub-band signal synthesis will increase the target signal processing gain and the signal to interference ratio will increase.

This paper is organized as follows: Section 2 presents the principle of range direction MIMO SAR. Section 3 proposes the pre-processing method of the concurrently transmitted sub-band signals of range direction MIMO SAR. Section 4 analyzes the efficiency of the proposed retransmission deception jamming suppression. Section 5 shows the results of our simulations. Section 6 concludes the paper.

## 2. Principle of Range Multiple Channel MIMO SAR

### 2.1. Geometry Model

The antenna is staggered in a direction that is vertical with the azimuth direction, which is called the range direction. There are *M* transmitting and receiving sub antennas comprising the whole linear MIMO antenna array. The OFDM-LFM [18,20] is used in the Range MIMO SAR, and each sub-band has a one-to-one correspondence with the sub-antenna element, which means that the number of OFDM sub-band signals is determined by the sub-antenna element count.

**Figure 1:**
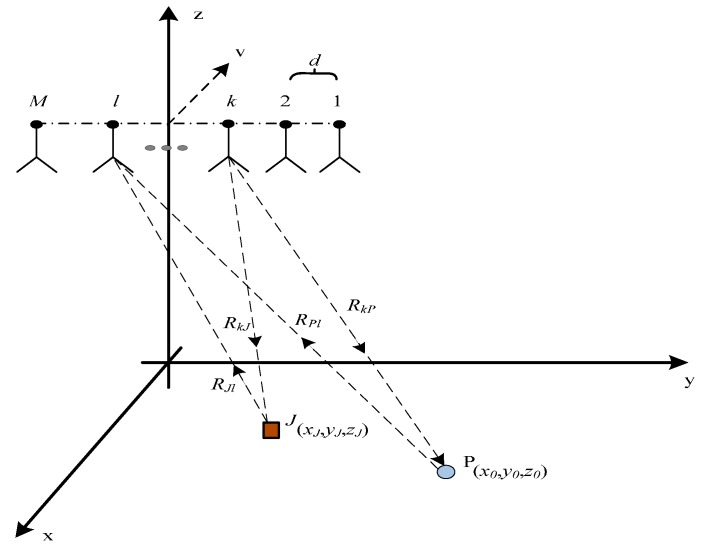
The Range MIMO SAR geometry model.

The geometry model is illustrated in Figure 1. We suppose that there are *M* sub-antenna elements, that the distance between them is equal, and that each antenna element can receive the transmitted signals from the others. As shown in Figure 1, we assume that the MIMO antenna linear array is along the *y*-axis. Its length is *L*, and the interval between each element is *d*. The MIMO SAR travels along with the *x*-axis with a speed of *v*. The height of the MIMO SAR is *H*. The projection of the SAR platform in the *xoy* plane is the coordinate origin. The range direction is along the *y*-axis. The middle point of the antenna *E_mid_* is located at (0, *y_m_*, 0) when the azimuth time is zero. Likewise, when the azimuth time is *t_a_*, the *k*-th antenna’s coordinate *E_k_* can be indicated as (*vt_a_*, *y_k_*, *H*). Hence, an arbitrary element *E_k_*’s *y*-axis coordinate is as follows:

(1) $$\begin{array}{cc} y_{k}=y_{m}+(k-\frac{1}{2}-\frac{M}{2})d & k=1\ldots M \end{array}$$

There is a scatter target and a jammer in the observation scene. Meanwhile, if the point scatter target *P* is located at (*x*_0_, *y*_0_, *z*_0_) and the jammer is located at (*x_J_*, *y_J_*, *z_J_*), the double trip instantaneous slant range of target *R_kl_*, which presents the slant range from the *k*-th transmitting antenna element to the *l*-th receiving antenna element, can be indicated as:

(2) $$\begin{array}{cc} R_{kl}(t_{a}) & =R_{kP}(t_{a})+R_{Pl}(t_{a})\\ & =\sqrt{{\left(vt_{a}-x_{0}\right)}^{2}+{\left(y_{k}-y_{0}\right)}^{2}+{\left(H-z_{0}\right)}^{2}}\\ & +\sqrt{{\left(vt_{a}-x_{0}\right)}^{2}+{\left(y_{l}-y_{0}\right)}^{2}+{\left(H-z_{0}\right)}^{2}} \end{array}$$

where, *t_a_* is the azimuth time, and *R_kP_* represents the single trip slant range from the *k*-th transmitting antenna to the target *P*. Similarly, *R_Pl_* is the single trip slant range from target *P* to the *l*-th receiving antenna. Likewise, the double trip instantaneous slant range of the jammer is:

(3) $$\begin{array}{cc} R_{kl}^{J}(t_{a}) & =R_{kJ}(t_{a})+R_{Jl}(t_{a})\\ & =\sqrt{{\left(vt_{a}-x_{J}\right)}^{2}+{\left(y_{k}-y_{J}\right)}^{2}+{\left(H-z_{J}\right)}^{2}}\\ & +\sqrt{{\left(vt_{a}-x_{J}\right)}^{2}+{\left(y_{l}-y_{J}\right)}^{2}+{\left(H-z_{J}\right)}^{2}} \end{array}$$

### 2.2. Signal Model

**Figure 2:**
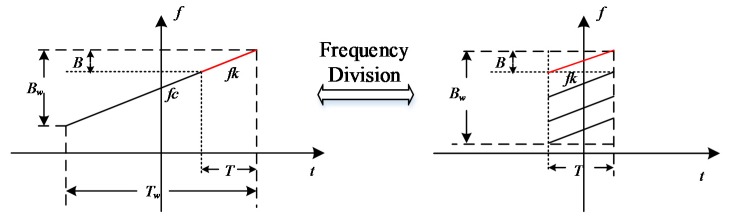
The illustration of the frequency dividing method.

Usually, the sub-band signal can be formed by dividing the wide band signal in the frequency domain. This is illustrated in Figure 2. The bandwidth of the original wide band signal is *B_w_,* and it is divided by the bandwidth *B*. After a time shift, the sub-band signal will be concurrently transmitted in the corresponding transmission channel.

Supposed that the pulse width is *T_w_*, the bandwidth is *B_w_*, the center frequency is *f_c_*, and the center time *T_c_* is 0. Therefore, the modulated frequency ratio *K_r_* is equal to *B_w_*/*T_w_*. *A_t_* is the signal amplitude. Then, the wideband signal can be indicated as:

(4) $$S_{w}(t)=A_{t}rect(\frac{t}{T_{w}})\text{exp}\left(j(2\pi f_{c}t+\pi K_{r}t^{2})\right)$$

If the wideband signal *S_w_* is divided into *M* sub-bands, the bandwidth of the sub-band signal *B* is *B_w_*/*M* and the pulse width of the sub-band signal *T* is *T_w_*/*M*. Therefore, the center frequency of the *k*-th sub-band signal is as follows:

(5) $$\begin{array}{cc} f_{k}=f_{c}+(k-\frac{1}{2}-\frac{N}{2})B & k=1\ldots M \end{array}$$

Then, the center time of the *k*-th sub-band signal is indicated as follows:

(6) $$\begin{array}{cc} T_{k}=T_{c}+(k-\frac{1}{2}-\frac{N}{2})T & k=1\ldots M \end{array}$$

So that the *k*-th sub-band transmitting signal with random phase modulation can be indicated as:

(7) $$S_{k}(t)=A_{t}rect(\frac{t}{T})\text{exp}\left(j(2\pi f_{k}t+\pi K_{r}t^{2}+\varphi_{k})\right)$$

Without consideration of the time delay, the concurrent transmission of all sub-band signals is as follows:

(8) $$S_{CT}(t)=A_{t}\sum_{k=1}^{M}rect(\frac{t}{T})\text{exp}\left(j(2\pi f_{k}t+\pi K_{r}t^{2}+\varphi_{k})\right)$$

We suppose that there are *n_c_* targets in the scene. Then, the target echoes, which are transmitted by the *k*-th antenna and received by the *l*-th antenna, can be indicated as:

(9) $$S_{kl}(t_{r},t_{a})=\sum_{n_{c}}A_{r}(\delta_{kl}^{n_{c}},R_{kl}^{n_{c}})rect(\frac{t_{r}-\frac{R_{kl}^{n_{c}}(t_{a})}{c}}{T})rect(\frac{t_{a}}{T_{a}})\text{exp}\left(j(2\pi f_{k}(t_{r}-\frac{R_{kl}^{n_{c}}(t_{a})}{c})+\pi K_{r}{\left(t_{r}-\frac{R_{kl}^{n_{c}}(t_{a})}{c}\right)}^{2}+\varphi_{k}(t_{a}))\right)$$

where *t_r_* and *t_a_* are the time of range direction and azimuth, respectively. *T* is the range direction time width of the sub-band signal. *T_a_* is the azimuth synthesis time. *f_k_* is the *k*-th working center frequency of the *k* th transmitting antenna. $\delta_{kl}^{n_{c}}$ is the *n_c_*-th target radar cross-section (RCS). *A_r_* is the receiving signal amplitude which is attenuated by the radar equation. Theoretically, the antenna elements corresponding with the sub-band will receive other antenna-transmitted sub-band signals because the frequency band of the antenna elements is equal to the wide band signal that is synthesized by the sub-band signal. Hence, the whole echoes of target signal are as follows:

(10) $$S(t_{r},t_{a})=\sum_{l}S_{l}(t_{r},t_{a})=\sum_{l}\sum_{k}S_{kl}(t_{r},t_{a})$$

Usually, we assume that the jammer can only intercept the signal located in its working frequency channel and accurately estimate all the parameters. According to the theoretical model of retransmission deception jamming [3,5], the major procedures are time delay and false information modulation. If the jammer intercepts the *I*-th sub-band signal, the deception jamming signal model is as follows:

(11) $$J(t_{r},t_{a})=\sum_{l}\sum_{n}A_{J}[\delta_{n}(t_{r}-(\frac{2\Delta R_{JP_{n}}(t_{a})}{c}-{\tau_{s}}^{\prime })-\tau_{s})\times S_{Il}(t_{r},t_{a})]\times \text{exp}(-j4\pi \frac{\Delta R_{JP_{n}}(t_{a})}{c})$$

where the $\Delta R_{JP_{n}}(t_{a})$ is the instantaneous single-trip slant range from point *J* to the *n*-th false target *P*, and $\tau_{s}$ is the system inherent time delay of the jammer. *A_J_* is the jamming amplitude. The operator * represents the convolution, which means the time delay of the intercepted signal. The false information is included in the additional phase modulation. Generally, the system time delay is beyond the PRT. Therefore, ${\tau_{s}}^{\prime }$ is utilized to modify the system time delay as follows:

(12) $$\begin{array}{ccc} {\tau_{s}}^{\prime }=\text{mod}(\tau_{s}{)}_{PRT} & & \tau_{s}>PRT \end{array}$$

so that the echoes, which include the target signal and the retransmitted deception jamming signal, can be represented as:

(13) $$\begin{array}{cc} S_{A}(t_{r}, & t_{a})=S(t_{r},t_{a})+J(t_{r},t_{a})\\ & =\sum_{l}\sum_{k}S_{kl}(t_{r},t_{a})+\sum_{l}\sum_{n}A_{J}[\delta_{n}(t_{r}-(\frac{2\Delta R_{JP_{n}}(t_{a})}{c}-{\tau_{s}}^{\prime })-\tau_{s})\times S_{Il}(t_{r},t_{a})]\times \text{exp}(-j4\pi \frac{\Delta R_{JP_{n}}(t_{a})}{c}) \end{array}$$

## 3. Range MIMO Signal Processing

Due to the one-to-one correspondence between the sub-band signal and the antenna element, the range MIMO SAR antenna with *M* channels is equivalent to having the capability of using *M* sub-band signals [20]. Moreover, the geometry model of the range MIMO SAR antenna will determine the position of the equivalent phase center [21], which is established to solve the MIMO signal processing.

**Figure 3:**
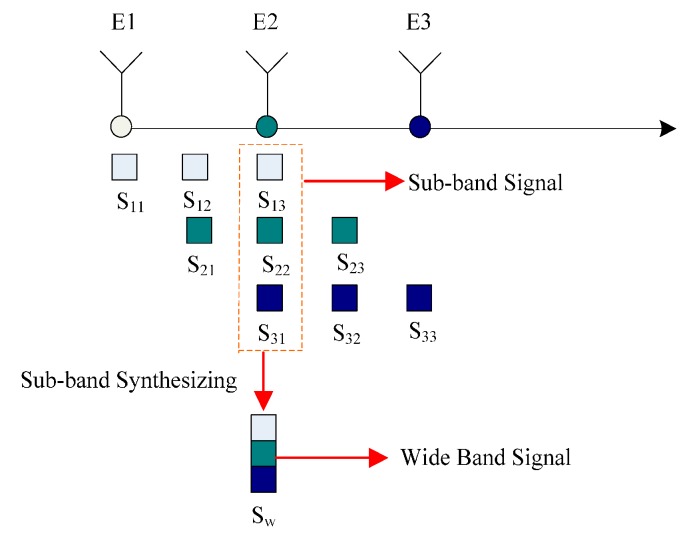
The equivalent phase center of the 3-channel range MIMO SAR.

The *M* antenna elements ranging along the *y*-axis will produce *M* × *M* equivalent phase centers, where (2 × *M* − 1)’s positions are different from each other. Hence, as Figure 3 shows, the 3-channel MIMO SAR has nine phase centers in total, and only five positions are independent. The equivalent phase center of the *k*-th transmitting antenna and *l*-th receiving antenna is located at *y_kl_*, and indicated by:

(14) $$y_{kl}=\left(y_{k}+y_{l}\right)/2$$

From the equation, the location of *y_kl_* is the same with *y_lk_*. As the figure shows, the equivalent phase center position of three transmitting elements is overlapped and situated in the 2nd antenna element position. In the overlapped phase center, the wide band signal will be synthesized without any additional conditions because the OFDM signal is utilized as the sub-band signal.

The bandwidth of the synthesized signal in the 2nd antenna elements is triple that of the sub-band signal when the bandwidth of the sub-band signal is equal. The adjacent phase center will synthesize twice the original band width of the sub-band signal. Simultaneously, the range direction resolution of each phase center is diverse, which is positively related to the bandwidth of the synthesized wide band signal.

Meanwhile, for the range direction MIMO SAR with *M* transmitting and receiving antenna channels, the bandwidth range of the synthesized signal is from 1 to *M* times the sub-band signal. To get the highest resolution and largest bandwidth, the middle equivalent phase center is utilized as an effective jamming suppression terminal. Range direction MIMO SAR has advantages compared to azimuth MIMO [20,22]. The merit of range MIMO is that the azimuth processing is equivalent to a traditional single channel SAR without consideration of non-uniform sampling and any Doppler ambiguity in azimuth direction [23,24].

The movement of every equivalent phase center is translational after an azimuth interval. Hence, the azimuth sample will not be staggered. According to the operation principle of equivalent phase center, the sum of the single-trip slant range *R_kP_* and *R_Pl_*, which is the actual range *R_kl_*, is approximate to the double-trip slant range ${\bar{R}}_{kl}$ from the equivalent phase center *E_kl_* with coordinate (*vt_a_*, *y_kl_*, *H*) to the target *P*:

(15) $${\bar{R}}_{kl}(t_{a})=2\sqrt{{\left(vt_{a}-x_{0}\right)}^{2}+{\left(y_{kl}-y_{0}\right)}^{2}+{\left(H-z_{0}\right)}^{2}}$$

The slant range *R_kl_* from the *k*-th transmitting antenna element *E_k_* to the *l*-th receiving antenna element *E_l_* is approximately equal to the double-trip slant range ${\bar{R}}_{kl}$ from the equivalent phase center *E_kl_* to the target *P*. For any antenna elements *E* with coordinate (*x*, *y*, *H*), the Taylor series expansion of the single trip slant range *R*(*t_a_*, *y*) around arbitrary point *y_T_* is:

(16) $$\begin{array}{cc} R(y) & =\sqrt{{\left(x-x_{0}\right)}^{2}+{\left(y-y_{0}\right)}^{2}+{\left(H-z_{0}\right)}^{2}}\\ & \approx R(y_{T})+\frac{\left(y_{T}-y_{0}\right)}{R(y_{T})}(y-y_{T})+\frac{1}{2}\frac{R^{2}(y_{T})-{\left(y_{T}-y_{0}\right)}^{2}}{R^{3}(y_{T})}{\left(y-y_{T}\right)}^{2}\\ & \approx R(y_{T})+A(y-y_{T})+B{\left(y-y_{T}\right)}^{2} \end{array}$$

Usually, the slant range *R* is expanded around *E_mid_*, i.e., the center point of the linear antenna array. Hence, Equation (2) can be written as follows:

(17) $$R_{kl}(y_{k},y_{l})\approx 2R(y_{m})+A\left(\left(y_{k}-y_{m})+(y_{l}-y_{m}\right)\right)+B\left({\left(y_{k}-y_{m}\right)}^{2}+{\left(y_{l}-y_{m}\right)}^{2}\right)$$

The position of the equivalent phase center is the center between the transmitting and receiving antenna elements. The Taylor series expansion of the equivalent phase center can be indicated as follows:

(18) $${\bar{R}}_{kl}(y_{kl})\approx 2R(y_{m})+2A(y_{kl}-y_{m})+2B{\left(y_{kl}-y_{m}\right)}^{2}$$

Meanwhile, the slant range difference is as follows:

(19) $$\Delta R=R_{kl}-{\bar{R}}_{kl}\approx B\frac{{\left(y_{k}-y_{l}\right)}^{2}}{2}$$

### 3.1. Phase Error Correction

The phase difference, which is caused by the range difference and has some negative effects on the SAR imaging, needs to be corrected in any imaging process. As Equation (16) shows, *B* can be indicated as:

(20) $$B=\frac{1}{2}\frac{R_{c⊥}^{2}(y_{m})}{R_{c}^{3}(y_{m})}$$

where *R_c_*(*y_m_*) presents the instantaneous slant range between the center point of the antenna array and the target point. $R_{c⊥}(y_{m})$ is the projection of *R_c_*(*y_m_*) in the *xoz* plane. Hence, the phase difference is as follows:

(21) $$\Delta \varphi =-\frac{\pi d_{kl}^{2}}{2\lambda }\frac{R_{c⊥}^{2}(y_{m})}{R_{c}^{3}(y_{m})}$$

where *d_kl_* = *y_k_* − *y_l_*. It presents the absolute interval between element *E_k_* and element *E_l_*.

On the one hand, the phase error is related to the synthesis aperture time for the same target. When the azimuth changed, the phase error for the same target *P* with coordinate (*x*_0_, *y*_0_, *z*_0_) can be indicated as:

(22) $$E_{\varphi t}=-\frac{\pi {d_{kl}}^{2}}{2\lambda }(\frac{R_{c⊥}^{2}(t_{a1})}{R_{c}^{3}(t_{a1})}-\frac{R_{c⊥}^{2}(t_{a2})}{R_{c}^{3}(t_{a2})})$$

**Figure 4:**
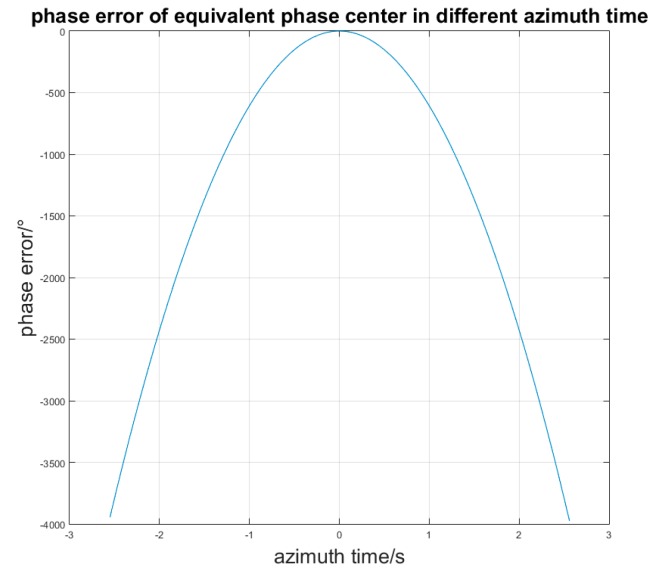
Phase error in different azimuth time.

We suppose that the velocity of the SAR platform is 300 m/s, *d_ij_* is 5 m, λ is 0.056 m, and the center point *P* is located at (0, 16010, 0). When *t_a_*_1_ is zero and *t_a_*_2_ is from −2.5/s to 2.5/s, the phase error change is as shown in Figure 4.

The phase error will increase when *t_a_*_2_ is far from *t_a_*_1_. Thus, the phase error needs to be corrected in each azimuth sampling time.

On the other hand, the phase error is related to the positions of the different targets in the main-lobe beam coverage. Usually, we use the slant range of the center point in the beam coverage to replace the slant ranges of the other targets in the scene. Meanwhile, the phase error will be imported. If there are two targets *P*_1_ and *P*_2_ with coordinates (*x*_1_, *y*_1_, *z*_1_) and (*x*_2_, *y*_2_, *z*_2_), the phase error between *P*_1_ and *P*_2_ is:

(23) $$E_{\varphi P}=-\frac{\pi {d_{kl}}^{2}}{2\lambda }(\frac{{\left(x-x_{1}\right)}^{2}+{\left(z-z_{1}\right)}^{2}}{{\left({\left(x-x_{1}\right)}^{2}+{\left(y-y_{1}\right)}^{2}+{\left(z-z_{1}\right)}^{2}\right)}^{3/2}}-\frac{{\left(x-x_{2}\right)}^{2}+{\left(z-z_{2}\right)}^{2}}{{\left({\left(x-x_{2}\right)}^{2}+{\left(y-y_{2}\right)}^{2}+{\left(z-z_{2}\right)}^{2}\right)}^{3/2}})$$

**Figure 5:**
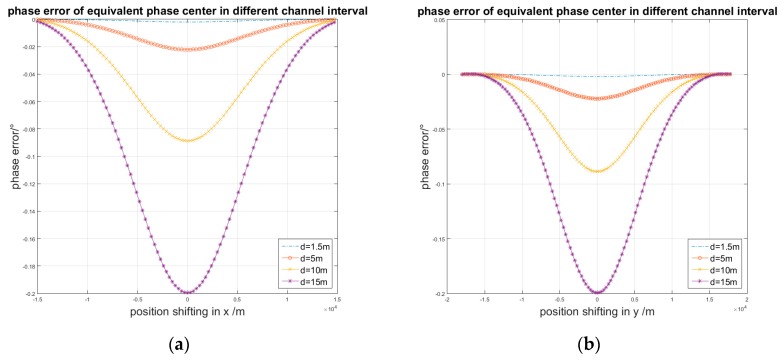
Phase error in different channel interval: (**a**) P2’s position shifting along the *x*-axis; (**b**) P2’s position shifting along the *y*-axis.

When the wave length and antenna interval are fixed, the phase error is only affected by the target position. We suppose that the position of target *P*_2_ is moving along the *x*-axis and *y*-axis. Then, *P*_1_ is located at (0, 0). Accordingly, for different intervals of the antenna channel *d*, the phase error is as shown by Figure 5.

**Figure 6:**
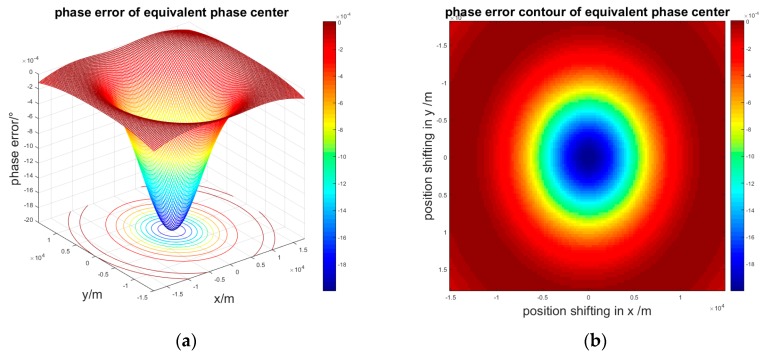
Phase error of the entire scene: (**a**) Three-dimensional phase error; (**b**) Contour of the phase error.

If target *P*_2_ is at any point in the scene and *d* is 1.5 m, the phase error of the beam coverage will be obtained as in Figure 6.

To obtain a high resolution image, the phase difference needs to be modified by using the center point of the beam coverage. The simulation proved that when the antenna interval *d* is smaller, the phase error is acceptable for image processing.

### 3.2. Signal Processing Flowchart

The signal processing includes two primary parts: preprocessing and imaging processing. After preprocessing, the traditional imaging algorithm can be utilized. The procedure of preprocessing is as follows: the first step in multiple sub-band signal processing is using the corresponding center frequency to demodulate the sub-band signals. Then, the sub-band signals are filtered to reduce the out-band noise and wide band jamming. The second step of the filtered sub-band signal is modifying the random phase of the sub-band signal. After this step, the target signal will be accurately matched. However, the system time delay of the deception jamming permits the jamming signal mismatch. The third step of the echo is the phase difference compensation for the multiple band signals. The fourth step of the compensated signal is the synthesis of the wide band signal using the sub-band signals. We supposed that there are *M* receiving and transmitting channels in the range MIMO SAR. Hence, the receiving signal can be indicated as:

(24) $$S_{r}=\left[\begin{array}{ccccccc} S_{11} & S_{12} & \cdots & S_{1M} & & & \\ & S_{21} & S_{22} & \cdots & S_{2M} & & \\ & & \ddots & \ddots & \cdots & \ddots & \\ & & & S_{M1} & S_{M2} & & S_{MM} \end{array}\right]$$

According to the position of the signal equivalent phase center, the sub-band signal is settled in the matrix. Every column presents a phase center. Meanwhile, the *Q*-th equivalent phase center signal is:

(25) $$\begin{array}{cc} S_{Q}^{E}=\sum_{k=1}^{Q}S_{k(Q+1-k)}(t_{r},t_{a}) & Q\in [1,2M-1] \end{array}$$

The band width is determined by the position of the equivalent phase center. *B_Q_*, the band width of $S_{Q}^{E}$, is as follows:

(26) $$B_{Q}=\left\{ \begin{array}{ll} QB & Q\leq M\\ \left(2M-Q\right)B & Q>M \end{array}\right.$$

Thus, the widest band width *B_W_* = *MB*. The number and bandwidth of the sub-band signal are two decisive components of *B_W_*. To analyze the preprocessing easily, we ignore the influence of the target number and RCS. Let $\tau_{a}=R_{ij}^{n_{c}}(t_{a})/c$, the equation can be indicated as:

(27) $$\begin{array}{cc} S_{Q}^{E}(t_{r},t_{a}) & =\sum_{k=1}^{Q}S_{k(Q+1-k)}(t_{r},t_{a})\\ & =\sum_{k=1}^{Q}rect(\frac{t_{r}-\tau_{a}}{T})rect(\frac{t_{a}}{T_{a}})\text{exp}\left(j(2\pi f_{k}(t_{r}-\tau_{a})+\pi K_{r}{\left(t_{r}-\tau_{a}\right)}^{2})\right) \end{array}$$

The *S_OB_*, echo of the transmitted wide band signal *S_w_*, is as follows:

(28) $$S_{OB}(t_{r},t_{a})=rect(\frac{t_{r}-\tau_{a}}{MT})rect(\frac{t_{a}}{T_{a}})\text{exp}\left(j(2\pi f_{c}(t_{r}-\tau_{a})+\pi K_{r}{\left(t_{r}-\tau_{a}\right)}^{2})\right)$$

Simultaneously, it can be represented by the sub-band signal $S_{Q}^{E}$.

(29) $$S_{OB}={S_{Q}^{E}|}_{Q=M}(t_{r}-(T_{k}-T_{c}))=\sum_{k=1}^{M}S_{k(M+1-k)}(t_{r}-\Delta T_{k},t_{a})$$

where $\Delta T_{k}$ is equal to *T_k_* − *T_c_*. The sub-band signal will be separated according to different carrying frequencies. Therefore, the sub-band signal is demodulated by the center frequency and compensated random phase:

(30) $$\begin{array}{cc} {S_{Q}^{E}|}_{Q=M} & =\sum_{k=1}^{M}S_{k(M+1-k)}(t_{r},t_{a})\text{exp}(-j2\pi f_{k}t_{r})exp(-j\varphi_{k}(t_{a}))\\ & =\sum_{k=1}^{M}rect(\frac{t_{r}-\tau_{a}}{T})rect(\frac{t_{a}}{T_{a}})\text{exp}\left(j(-2\pi f_{k}\tau_{a}+\pi K_{r}{\left(t_{r}-\tau_{a}\right)}^{2})\right) \end{array}$$

Then, every sub-band signal in the same equivalent phase center is time shifted. Let $t_{r}=t_{r}-\Delta T_{k}$, so the process is as follows:

(31) $$\begin{array}{cc} {S_{Q}^{E}|}_{Q=M}(t_{r}-\Delta T_{k}) & =\sum_{k=1}^{M}rect(\frac{t_{r}-\Delta T_{k}-\tau_{a}}{T})rect(\frac{t_{a}}{T_{a}})\text{exp}\left(j(-2\pi f_{k}\tau_{a}+\pi K_{r}{\left(t_{r}-\Delta T_{k}-\tau_{a}\right)}^{2})\right)\\ & =\sum_{k=1}^{M}rect(\frac{t_{r}-\tau_{a}}{MT})rect(\frac{t_{a}}{T_{a}})\text{exp}\left(j(-2\pi f_{c}\tau_{a}+\pi K_{r}{\left(t_{r}-\tau_{a}\right)}^{2})\right)\text{exp}(-j2\pi \Delta f_{k}t_{r})\text{exp}(j\pi K_{r}\Delta {T_{k}}^{2}) \end{array}$$

**Figure 7:**
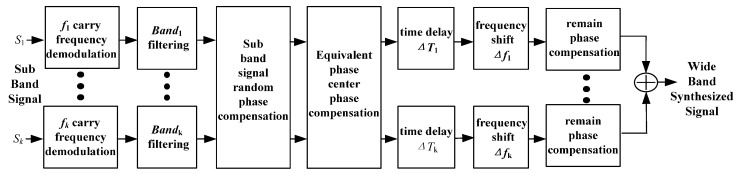
Range MIMO SAR signal preprocessing flowchart.

Comparing this equation with Equation (30), the time shifted sub-band signal lacks a linear phase and a constant phase. The frequency shift and phase compensation will recover the wide band by using the time-shifting sub-band signals. In conclusion, the signal preprocessing flow chart of range MIMO SAR which adopts the OFDM signal as the sub-band signal and random phase modulation, is as seen in Figure 7.

After preprocessing, the sub-band signals have to be synthesized into a wide band signal that can be utilized to obtain SAR images by a traditional imaging algorithm. Moreover, the preprocessing will suppress the jamming signals.

## 4. Range Direction MIMO SAR Anti-Deception Jamming Efficiency Analysis

**Figure 8:**
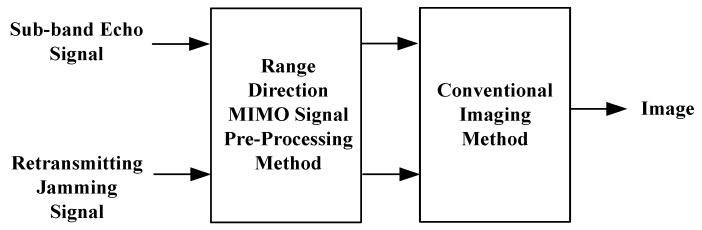
Anti-jamming efficiency analysis flowchart.

Considering the retransmision deception jamming in the echoes as Figure 8, the anti-jamming efficiency of the range MIMO SAR is analyzed after signal pre-processing and imaging.

Because the range direction MIMO SAR using the concurrently transmitted sub-band signal is modulated by a random phase, the jammer processing time will increase and even cannot intercept and recognize concurrently transmitted signals. The inherent system time delay $\tau_{s}$ can be indicated as follows:

(32) $$\tau_{s}=\left\lfloor \tau_{s}/PRT\right\rfloor PRT+\text{mod}(\tau_{s}{)}_{PRT}=nPRT+{\tau_{s}}^{\prime }$$

where ⌊ ⌋ represents selecting the lesser round number, and mod means selecting the remainder. The integer *n* indicates that the jamming signal will fall behind *n* PRTs in the SAR receiving terminal. As in Equation (11), we suppose that the jammer intercepts the *I*-th sub-band signal:

(33) $$J(t_{r},t_{a})=A_{J}S_{Il}^{J}(t_{r}-\frac{2\Delta R_{JP}(t_{a})}{c},t_{a}-nPRT)$$

After carrying out frequency demodulation and random phase demodulation, the deception jammer signal is as follows:

(34) $$\begin{array}{cc} J(t_{r},t_{a}) & =A_{J}rect(\frac{t_{r}-\tau^{\prime }}{T})rect(\frac{t_{a}}{T_{a}})\text{exp}\left(j\pi K_{r}{\left(t_{r}-\tau^{\prime }\right)}^{2}\right)\\ & \cdot \text{exp}(-j2\pi f_{k}\tau^{\prime })\text{exp}(j(\varphi_{I}(t_{a})-\varphi_{I}(t_{a}-nPRT))) \end{array}$$

where *A_m_* means the jammer signal gain [4,25]. $\tau^{\prime }$ is the time delay and is given as follows:

(35) $$\tau^{\prime }=\frac{R_{Il}^{J}(t_{a})+2\Delta R_{JP}(t_{a})}{c}$$

After range compression, the jammer signal is as follows:

(36) $$\begin{array}{cc} J(t_{r},t_{a}) & =A_{J}T\sin c(B_{r}(t_{r}-\tau^{\prime }))rect(\frac{t_{a}}{T_{a}})\text{exp}(-j2\pi f_{k}\tau^{\prime })\\ & \cdot \text{exp}(j\Delta \varphi (t_{a})) \end{array}$$

where $\Delta \varphi (t_{a})$ means the phase difference caused by the jammer system time delay and is equal to the minus phase:

(37) $$\Delta \varphi (t_{a})=\varphi_{I}(t_{a})-\varphi_{I}(t_{a}-nPRT)$$

Next, we suppose that the slant range of the deception target generated by the retransmit jamming is approximate with the real target:

(38) $$R_{Il}^{P}(t_{a})\approx R_{Il}^{J}(t_{a})+2\Delta R_{JP}(t_{a})$$

Hence, after the Range Cell Migration Correction, the false target *P* will be corrected into the shortest slant range $R_{Il}^{P}$. The jamming signal is as follows:

(39) $$\begin{array}{cc} J(t_{r},t_{a}) & =A_{J}T\sin c(B_{r}(t_{r}-\frac{R_{Il}^{P}}{c}))rect(\frac{t_{a}}{T_{a}})\text{exp}(-j2\pi f_{k}\tau^{\prime })\\ & \cdot \text{exp}(j\Delta \varphi (t_{a})) \end{array}$$

If the azimuth compression response function is $h_{a}(t_{a})$, the azimuth compression process is as follows:

(40) $$\begin{array}{cc} J(t_{r},t_{a}) & =\int_{T_{a}}rect(\frac{\tau }{T_{a}})\text{exp}(-j2\pi f_{k}(\alpha \tau +\beta \tau^{2}))\text{exp}(j\Delta \varphi (\tau ))h_{a}(\tau -t_{a})d\tau \\ & \cdot A_{J}T\sin c(B_{r}(t_{r}-\frac{R_{Ij}^{P}}{c})) \end{array}$$

Based on the integral mean value theorem, the azimuth gain of the jamming signal is in the range given by the expression:

(41) $$T_{a}\text{min}(J_{a}(\tau )h_{a}(\tau -t_{a}))\leq \int_{T_{a}}J_{a}(\tau )h_{a}(\tau -t_{a})d\tau <T_{a}\text{max}(J_{a}(\tau )h_{a}(\tau -t_{a}))$$

where *J_a_* is the azimuth signal of the entire jamming signal *J*. Equation (41) shows that the jamming signal defocuses in the azimuth direction due to the random phase mismatch. The greater the phase difference randomness, the lower the jamming signal pulse compression processing gain. The shorter the integrating range, the lower the jamming signal compression processing gain. We suppose that the jamming signal azimuth processing gain is *K* (*T_a_*), which is less than the real target azimuth processing gain *T_a_*. As Equation (40) shows, the jamming signal will achieve sub-band range compression gain. Hence, according to whole signal processing flowchart, the total gain of the jamming signal is proportional to the following equation:

(42) $$G_{JP}\propto A_{J}TK(T_{a})$$

As for the single sub-band target signal, after the range and azimuth compression processing, the total gain is as follows:

(43) $$G_{Psig}\propto TT_{a}$$

Then, the sub-band signal will be synthesized to a wide band signal, which will increase the range processing gain. The total gain will be as follows:

(44) $$G_{Pall}\propto MTT_{a}$$

Hence, the peak aptitude ratio of the target to jamming without sub-band synthesizing is indicated as:

(45) $$r_{A1}=\frac{G_{Psig}}{G_{JP}}=\frac{T_{a}}{A_{J}K(T_{a})}$$

The peak aptitude ratio of target to jamming after sub-band synthesizing is indicated as:

(46) $$r_{A2}=\frac{G_{Pall}}{G_{JP}}=\frac{MT_{a}}{A_{J}K(T_{a})}=Mr_{A1}$$

## 5. Simulation

Retransmission deception jamming is simulated in this part. We supposed that the range direction MIMO SAR has 5-channel antenna elements. The geometry model is as shown in Figure 1. The target is located in a 3 × 3 matrix that has a middle point *P* in the center of the scene with coordinates (0, 16010, 0). Then, the interval in the range direction of the point matrix is 10 m, and the interval in the azimuth is 30 m. The jammer *J* is located in the adjacent point in the same row with coordinates (0, 16000, 0) in the point matrix. The other simulation conditions are given the Table 1.

**Figure 9:**
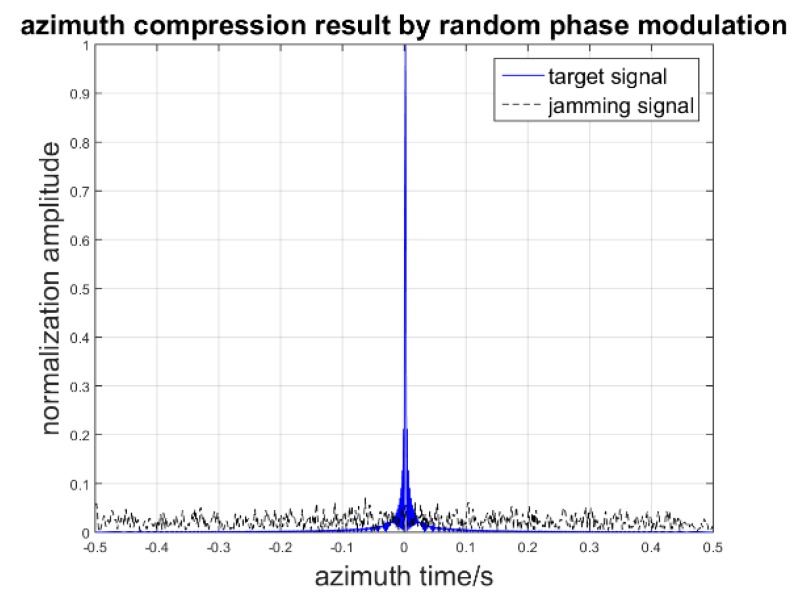
Azimuth compression result of target and jamming.

**Figure 10:**
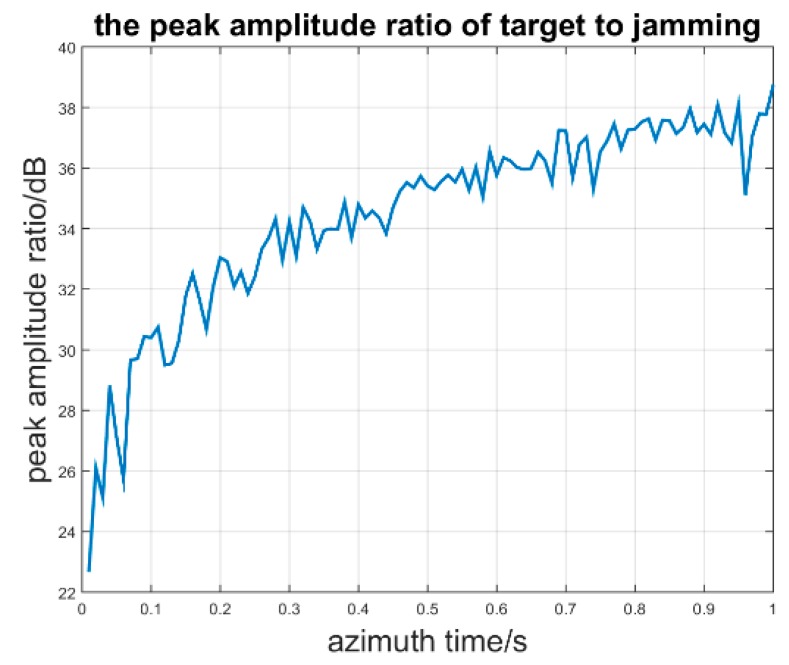
Peak amplitude ratio in different azimuth time.

First, the random phase modulation is simulated. We suppose that the modulation random phase obeys Gaussian noise N(0,2π) and that the jammer system time delay is 2 PRTs. When the azimuth synthesis time and frequency modulation rate are fixed such that the azimuth time is 1 s and the chirp rate in the azimuth direction is 500 Hz, the result of the jammer signal and target signal azimuth compression processing is as follows: Figure 9 shows that the deception jamming signal will defocus after the azimuth compression because of the phase residue in random phase compensation. The phase residue is associated with the time delay of retransmitting jamming signal. Next, we analyze the effect of the azimuth time to the peak amplitude ratio (PAR) of the target signal to the jamming signal without sub-band synthesis. The Monte-Carlo experiment is simulated when the azimuth chirp rate is 50 kHz/s and the sampling rate is 60 kHz/s. The azimuth time is from 0.01 s to 1 s, and the random phase still obeys N(0,2π). The peak amplitude ratio simulation is as seen in Figure 10.

**Figure 11:**
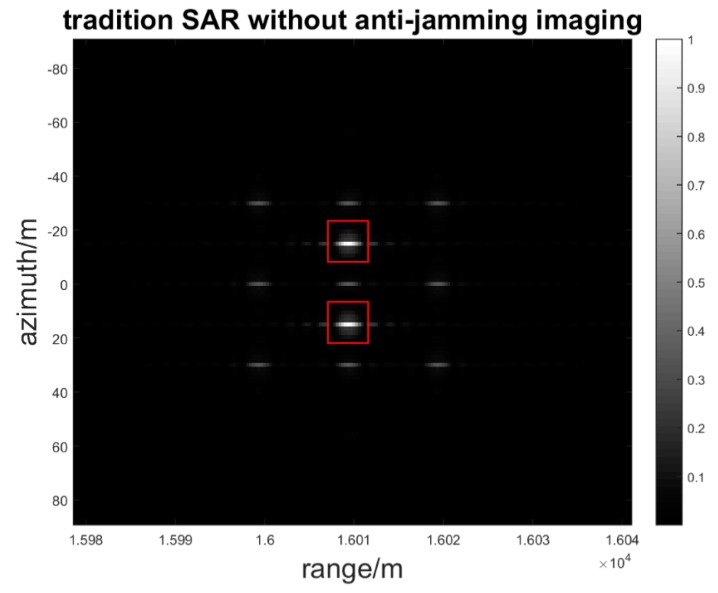
Deception jamming image in traditional SAR.

It is noticeable that the PAR will increase when the azimuth time is increasing, which is consistent with the theoretical analysis that *r_A1_* is proportional to the azimuth time. The effect of the random phase modulation relies on the azimuth time. The longer the azimuth time, the better the jamming suppression result. Second, we suppose that the inherent jammer delay time is 2 PRT and that the jammer intercepts the third band signal and forms the retransmitted jamming signal. When the jammer *J* forms two false point targets *P_F_*_1_ and *P_F_*_2_ located at (15, 16010, 0) and (−15, 16010, 0), respectively, the imaging result is as seen in Figure 11.

When the interference to signal ratio is from 0 dB to 40 dB and the step is 20 dB, which means that the interference power is increasing, the sub-band synthesis processing of range MIMO SAR and single band processing are simulated, respectively.

**Figure 12:**
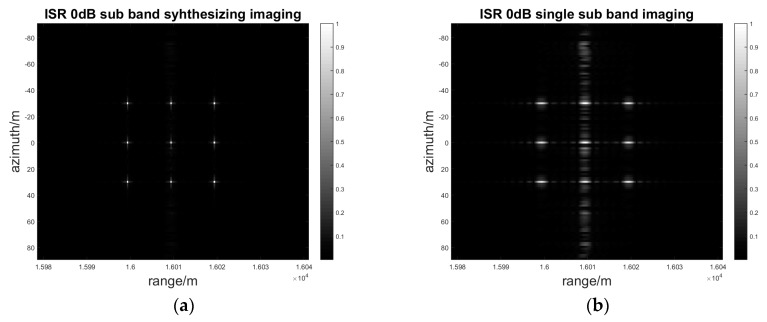
ISR 0 dB sub-band synthesizing and single sub-band imaging result: (**a**) Sub-band synthesis of a two-dimensional gray image; (**b**) Single sub-band of a two-dimensional gray image.

**Figure 13:**
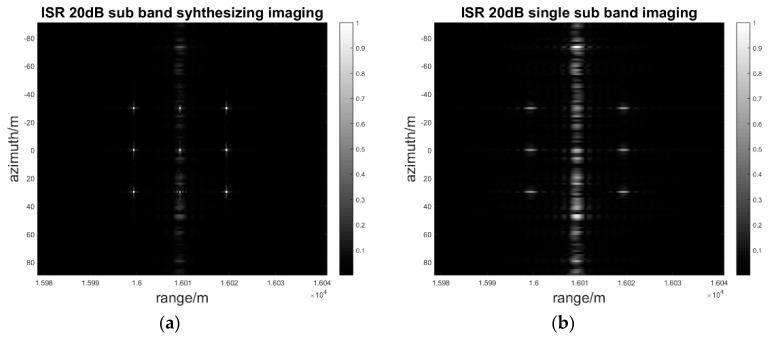
ISR 20 dB sub-band synthesis and single sub-band imaging result: (**a**) Sub-band synthesis of a two-dimensional gray image; (**b**) Single sub-band of a two-dimensional gray image.

**Figure 14:**
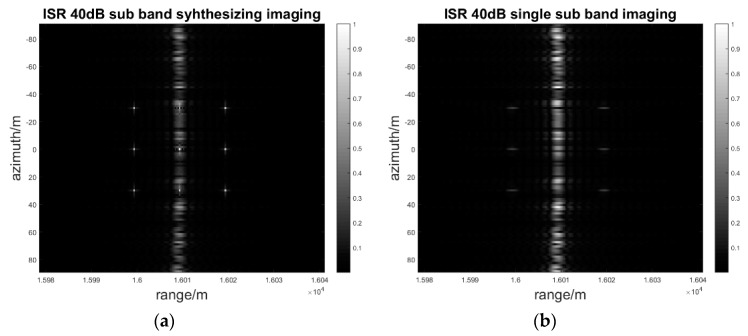
ISR 40 dB sub-band synthesis and single sub-band imaging result: (**a**) Sub-band synthesis of a two-dimensional gray image; (**b**) Single sub-band synthesis of a two-dimensional gray image.

As Figure 12, Figure 13 and Figure 14 show, the deception jamming signal is obviously defocusing in the azimuth in both sub-band synthesis and single band processing so that there is a jamming stripe forming along the azimuth. Owing to the random phase modulation, the deception jamming only gets the range direction compression gain. Meanwhile, the jamming signal is located in the right range bin with the range coordinate 16010.

As the jamming power increases from 0 dB to 40 dB, the amplitude of the jamming stripe also increases. With the same jamming power, the jamming suppression effect of sub-band synthesis processing is better than that of the single sub-band processing. As Figure 14b shows, the amplitude of jamming is greater than that of the real target in single sub-band processing, which has negative effects on the image interpretation. Nevertheless, as Figure 14a shows, the amplitude of the jamming signal is still less than that of the real target in sub-band synthesis processing, which almost has no negative effects on the image interpretation. The quantitative results for the SIR and PAR of the signal to interference is indicated in Table 2.

**Figure 15:**
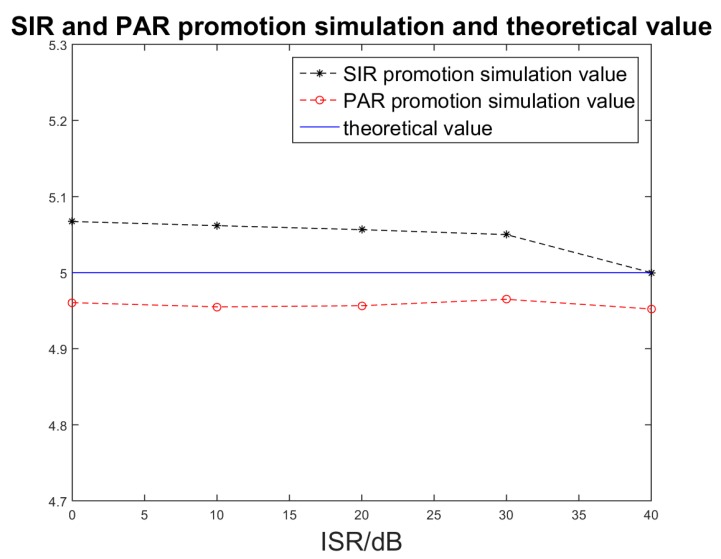
SIR and PAR promotion simulated and theoretical value.

As Table 2 shows, after image processing, the SIR and PAR, including the sub-band synthesis process and the single sub-band process, will decrease when the ISR in the echoes is increasing. However, the SIR and PAR of the sub-band synthesis process are higher than those of the single band process, which has also been illustrated in Figure 12, Figure 13 and Figure 14. Even when the deception jamming signal power is 40 dB, the PAR of the sub-band signal synthesis is 2.9. Nevertheless, the PAR of the single sub-band process is 0.59, which means the amplitude of the jamming signal is larger than what the target shows in Figure 14b. After sub-band synthesis is used, the SIR and PAR will have increases in the sub-band number of times in the theoretical analysis. The simulation result is as follows:

As Figure 15 shows, the PAR promotion *r*_A2_/*r*_A1_ and SIR promotion are both approximately 5, which is the sub-band number in the simulation. Hence, by comparing the results of sub-band synthesis processing and single sub-band processing, one obvious feature is that the range direction MIMO SAR can effectively suppress deception jamming.

## 6. Conclusions

In this paper, a range direction MIMO SAR system is proposed as a novel retransmission deception jamming suppression SAR. Based on concurrent transmission of sub-band signals with random initial phase modulation and sub-band synthesis in the equivalent phase center of the MIMO SAR, the jamming suppression capability for retransmission deception jamming is enhanced.

First, the sub-band signal synthesis processing model is built. Then, the efficiency of deception jamming suppression is analyzed. Because of the combination of the sub-band synthesis and random phase modulation, the retransmission deception jamming will defocus in the azimuth, and the interference to signal ratio will decrease. Finally, the simulation of deception jamming proves the validity of the jamming suppression method. Hence, the MIMO SAR will not only improve the spatial resolution and wider swath coverage, but will also enhance the jamming suppression capability. In the future, we will investigate non-coherent integration of different equivalent phase centers in the image domain to further improve the anti-jamming capability of the proposed method.

## Figures and Tables

**Table 1:** Simulation conditions.

Parameter	Value
Carry Frequency	5.3 GHz
Sub-band Number	5
Channel interval *d*	1.5 m
SAR speed *v*	300 m/s
SAR height *H*	8000 m
Pulse width of sub band signal *T*	5 µs
Chirp rate in range direction	20 × 10^12^ Hz/s
Over sampling rate	1.2
Band width in azimuth	80 Hz
Pulse Repetition Frequency	100 Hz

**Table 2:** The quantitative results for retransmitting deception jamming suppression.

ISR (dB)	SIR_2_^1^	SIR_1_^2^	*r*_*A*2_^3^	*r*_*A*1_^4^
0	5.7447	1.1337	43.5424	8.7777
10	1.8101	0.3576	16.4348	3.3168
20	0.5729	0.1133	11.8402	2.3888
30	0.1818	0.0360	7.7389	1.5587
40	0.0575	0.0115	2.9233	0.5903

^1^ SIR_2_: signal to interference ratio; ^2^ SIR_2_: single sub-band signal to interference ratio; ^3^ *r*_*A*2_: the peak aptitude ratio of sub-band synthesizing signal to interference; ^4^ *r*_*A*1_: the peak aptitude ratio of single sub-band signal to interference.
